# Concepts of Cardiac Dyssynchrony and Dynamic Approach

**DOI:** 10.3390/diagnostics14090937

**Published:** 2024-04-30

**Authors:** Bianca Iulia Catrina, Florina Batar, Ioan Manitiu, Liliana Prodan, Ciprian Tanasescu, Teodora Filip

**Affiliations:** 1 County Clinical Emergency Hospital of Sibiu, 550245 Sibiu, Romania; florina.batar@ulbsibiu.ro (F.B.); ioanmanitiu@yahoo.com (I.M.); tanasescuciprian@yahoo.fr (C.T.); filip.teodora96@gmail.com (T.F.); 2 Pathophysiology Department, Faculty of Medicine, Lucian Blaga University of Sibiu, 550169 Sibiu, Romania; 3 Physiology Pathophysiology Department, Faculty of Medicine, Lucian Blaga University of Sibiu, 550169 Sibiu, Romania; 4 Cardiology Department, Faculty of Medicine, Lucian Blaga University of Sibiu, 550169 Sibiu, Romania; 5 Clinical Surgical Department, Faculty of Medicine, Lucian Blaga University of Sibiu, 550169 Sibiu, Romania

**Keywords:** dyssynchrony, resynchronization, left bundle branch block, His, HOT-CRT

## Abstract

Cardiac conduction involves electrical activity from one myocyte to another, creating coordinated contractions in each. Disruptions in the conducting system, such as left bundle branch block (LBBB), can result in premature activation of specific regions of the heart, leading to heart failure and increased morbidity and mortality. Structural alterations in T-tubules and the sarcoplasmic reticulum can lead to dyssynchrony, a condition that can be treated by cardiac resynchronization therapy (CRT), which stands as a cornerstone in this pathology. The heterogeneity in patient responses underscored the necessity of improving the diagnostic approach. Vectocardiography, ultra-high-frequency ECG, 3D echocardiography, and electrocardiographic imaging seem to offer advanced precision in identifying optimal candidates for CRT in addition to the classic diagnostic methods. The advent of His bundle pacing and left bundle branch pacing further refined the approach in the treatment of dyssynchrony, offering more physiological pacing modalities that promise enhanced outcomes by maintaining or restoring the natural sequence of ventricular activation. HOT-CRT emerges as a pivotal innovation combining the benefits of CRT with the precision of His bundle or left bundle branch area pacing to optimize cardiac function in a subset of patients where traditional CRT might fall short.

## 1. Introduction

The movement of electrical activation from one myocyte to another, causing synchronized contractions in each, is known as cardiac conduction. The impulses that originate in the sinoatrial node propagate to the atria and then to the ventricles through the unique His–Purkinje conduction system. The sequential activation obtained is crucial in converting the longitudinal contraction of individual myocytes into the intricate three-dimensional pumping motion of the heart [1].

Disruptions in the conducting system, such as left bundle branch block, found in 30–50% of dilated cardiomyopathies [2,3], leads to an early stimulation of certain heart regions, which will determine early contraction at a reduced load [4]. Consequently, with this, we witness failure to generate sufficient pressure to open the aortic valve and impart energy to the later-activated regions. Delayed contracting regions will stretch the earlier-stimulated ones, causing internal blood transfer and reducing myocardial work efficiency [5]. In the context of heart failure, ventricular dyssynchrony exacerbates both morbidity and mortality [6].

Dyssynchrony can also occur due to myocardial infarction, in which the afflicted area experiences reduced or obstructed conduction and impaired contractility. This can result in dyssynchrony of the left ventricle if the infarcted region is sufficiently extensive [7]. RV pacing can have a negative effect on LV performance due to the asynchronous activation of the myocardium, which can result in pacing-induced cardiomyopathy [8].

Ventricular dyssynchrony comprises two distinct components: mechanical dyssynchrony and electrical dyssynchrony. Electrical dyssynchrony is the term used to describe conduction delay that leads to a QRS duration exceeding 120 msec on the surface electrocardiogram. Conversely, mechanical dyssynchrony refers to the disparity in the timing of mechanical contraction and relaxation among the various segments of the left ventricle. This discrepancy is caused by electrical dyssynchrony [9,10].

**Figure 1:**
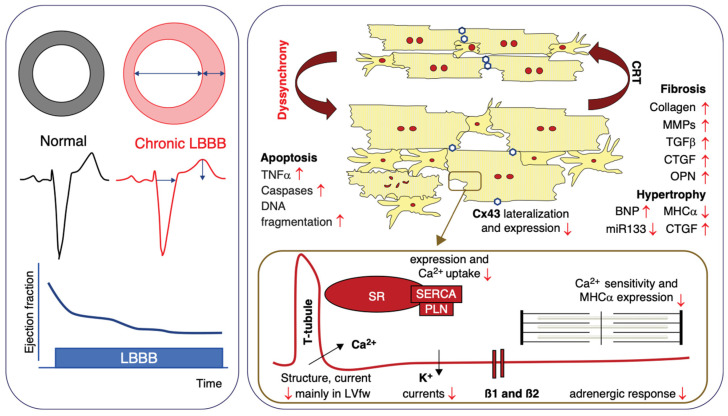
Processes contributing to structural, electrical, and contractile remodeling in the dyssynchronous heart, as seen on functional measurements (**left**) and on a cellular and molecular level (**right**) (Photo extracted from Kenneth A. Ellenbogen et al. The evolving state of cardiac resynchronization therapy and conduction system pacing: 25 years of research at EP Europace journal, *EP Europace*, Volume 25, Issue 8, August 2023, by permission of Oxford University Press).

On a cellular level, dyssynchrony involves structural changes in T-tubules and the sarcoplasmic reticulum, where the registration of ryanodine receptor and voltage-gated channels becomes disrupted, partially tackled by CRT as seen in Figure 1 [11]. Cardiomyocytes exhibit not only reduced strength in dyssynchronous heart failure but also distinct response to beta-adrenergic stimulation, whereas CRT enhances their strength and restores responsiveness to near normal [12]. The mechanism for restoring beta-adrenergic signaling highlights the importance of CRT by not just reestablishing electromechanical synchrony but also engaging the subcellular mechanism [13].

Heart failure frequently presents with an extended duration of action potential (APD), which is caused by anomalies in different membrane channels, such as a decrease in repolarizing potassium currents [14]. Dyssynchrony worsens this issue by introducing variability in APD in the late-activated lateral wall, and it is well established that extended APD promotes the occurrence of ventricular arrhythmias [15]. Moreover, persistent dyssynchrony disrupts the functioning of myofilaments by decreasing their ability to respond to calcium stimulation. Cardiac resynchronization therapy (CRT) can counteract this effect by completely restoring calcium sensitivity to its normal levels through increased phosphorylation [16].

Dyssynchrony also affects molecular metabolic remodeling, leading to regional disparities in glucose absorption [17], elevated basal mitochondrial oxygen consumption [18], and reduced ATPase activity [19].

## 2. Diagnostic Techniques

### 2.1. Electrocardiography

The application of twelve-lead electrocardiograms (ECGs) has provided significant knowledge regarding abnormalities in ventricular conduction. The recommendation for CRT implantation is given a class IA indication when the QRS duration exceeds 150 msec and a left bundle branch block (LBBB) morphology is observed [20]. However, QRS duration alone is insufficient for distinguishing between right- or left-sided conduction abnormalities and inter- versus intraventricular dyssynchrony. Therefore, QRS morphology provides supplementary information, but it is susceptible to subjective interpretation due to the existence of multiple definitions for specific conduction disturbances such as LBBB [21].

Recent years have seen the emergence of a number of non-invasive techniques that, by incorporating spatial and temporal data, permit a more precise examination of electrical dyssynchrony. Vectorcardiography and ultra-high frequency ECG are two techniques that are initially described in relation to the conventional twelve-lead ECG. By utilizing body surface potential measurements gated from 200 electrodes positioned around the chest and coupled with the patient-specific heart–torso geometry, a more advanced technology is capable of reconstructing the electro-anatomical activation of the epicardium [22].

### 2.2. Echocardiography

Merely relying on electrical dyssynchrony (QRS prolongation > 120 ms) appears insufficient in sensitivity to identify mechanical dyssynchrony, which may be more critical in nature. Additionally, QRS dispersion may outperform its duration, whereas marked QRS prolongation may be less predictive of response than intermediate QRS prolongation. Mechanical dyssynchrony, which is commonly categorized as follows, is thus directly addressed by echocardiography: (1) Atrioventricular refers to a crucial aspect of cardiac resynchronization therapy (CRT) optimization; (2) interventricular pertains to the space between the ventricles; (3) intraventricular refers to the delay in activation between different portions of the left ventricle (LV) [23].

**Figure 2:**
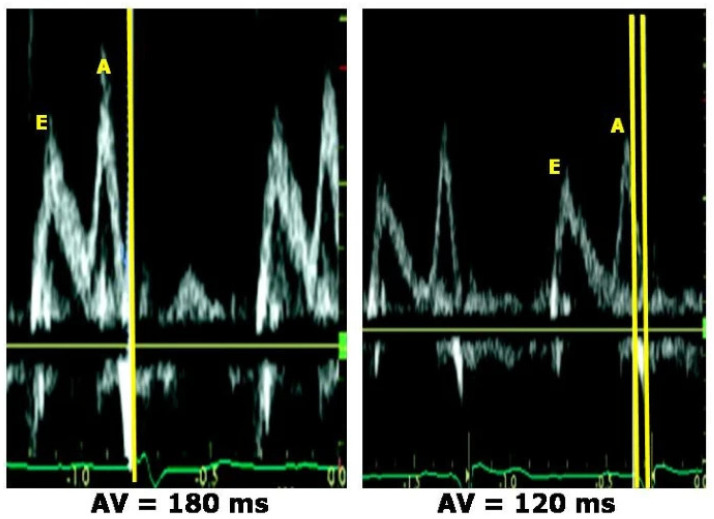
Optimization of AV delay after CRT by pulsed Doppler mitral inflow pattern. (Photo extracted from Galderisi, M. et al. Doppler echocardiography and myocardial dyssynchrony: a practical update of old and new ultrasound technologies. Cardiovasc Ultrasound 5, 28 (2007)) [25].

Delayed atrioventricular conduction can produce AV dyssynchrony, leading to mitral valve insufficiency. Attending to this topic is essential for maintaining optimal cardiac performance, as assessed using conventional Doppler measurements of mitral inflow E and A waves as seen in Figure 2 [24].

**Figure 3:**
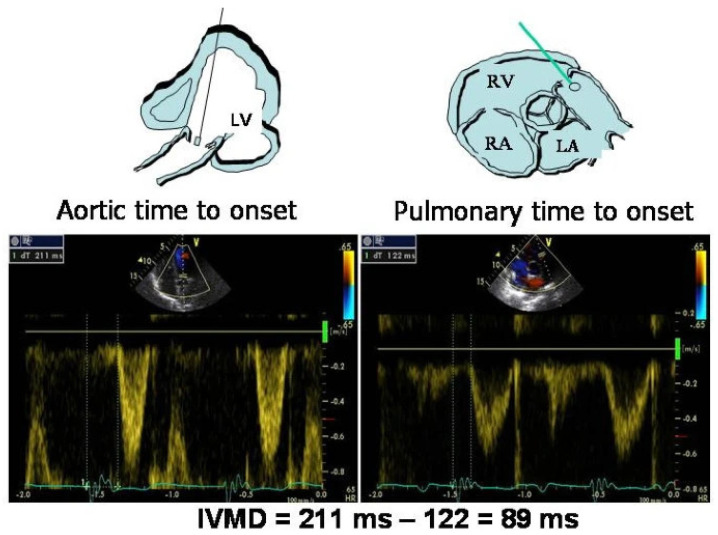
Estimation of interventricular mechanical delay using the conventional Doppler technique. The duration from the occurrence of the ECG Q wave to the onset of the right ventricular outflow tract is 122 ms, whereas the time from the Q wave to the onset of the left ventricular outflow tract is 211 ms (left panel). An interventricular mechanical delay (IVMD) of 89 milliseconds is obtained, which signifies the presence of substantial interventricular dyssynchrony (Photo extracted from Galderisi, M. et al. Doppler echocardiography and myocardial dyssynchrony: a practical update of old and new ultrasound technologies. Cardiovasc Ultrasound 5, 28 (2007) [25].

One method of evaluating interventricular dyssynchrony (IVD) is through the time difference between Doppler pulmonic and aortic outflow or by employing TDI with sample volumes from specific locations. Intraventricular dyssynchrony is defined as an activation delay between the RV and LV that exceeds 40 ms, as seen in Figure 3 [24].

**Figure 4:**
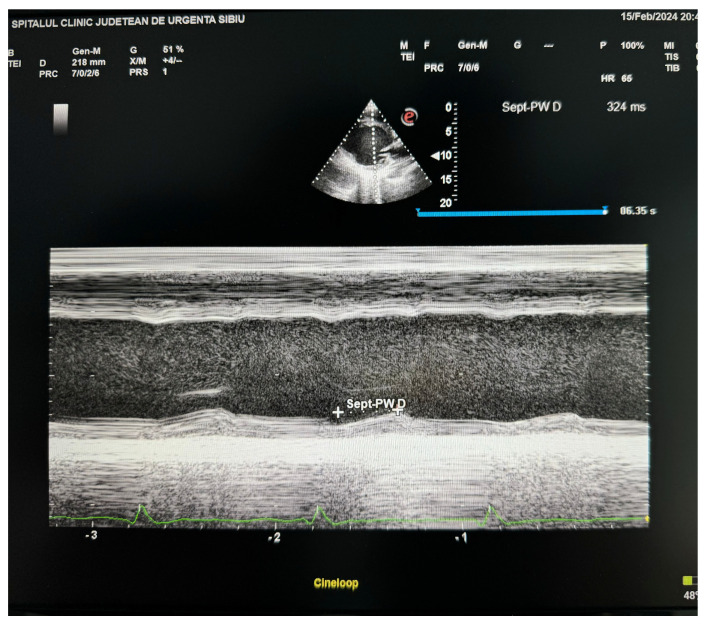
M-mode evaluation at midventricular level demonstrating a septal to posterior wall delay of 324 msec (from personal archive).

The M-mode technique, despite its simplicity, examines dyssynchrony via the septal and posterior walls. However, its applicability is restricted to two segments of the basal wall, and it encounters difficulties when attempting to assess mid-ventricular dyssynchrony or visualize peak wall motion, as seen in Figure 4 [24,26].

Tissue velocity imaging is the prevailing method utilized to assess intraventricular dyssynchrony, which refers to an irregular timing of motion of left ventricular (LV) walls during systole. This is facilitated by the capability of numerous ultrasound devices to measure myocardial velocities. The peak systolic velocity may be challenging to ascertain in particular patients, and the calculation process requires additional effort [25]. Tissue tracking is an additional method that demonstrates exceptional performance in quantifying regional and global longitudinal systolic function, minimizing observer bias, determining peak wall motion timing rapidly, and accurately assessing LV dyssynchrony; thus, it can detect subtle changes [26]. Technical aspects such as optimizing frame rate to minimize variability and guaranteeing consistent electrocardiogram (ECG) signals are critical, despite the clinical potential of tissue Doppler imaging. Challenges arise from analyzing data from patients not on sinus rhythm or from professionals having the proper training required [26].

Time-to-peak strain evaluation via speckle tracking echocardiography (STE) became the predominant method utilized to assess dyssynchrony following the PROSPECT trial. This approach allows for the evaluation of segmental myocardial deformation instead of mobility. One benefit of STE is its ability to address some limitations of TDI-based technologies, such as the tethering effect and angle dependency. This technique was considered to be crucial for assessing dyssynchrony. The approach that received the most attention and was published during this period was the time-to-peak septal to posterior wall radial strain delay method. By employing this method, it was determined that a delay threshold of 130 ms or less at baseline was correlated with a favorable prognosis following CRT [27].

The speckle-tracking algorithm, when used on routine gray-scale images, can accurately measure radial left ventricular (LV) dyssynchrony in patients with heart failure (HF). It can also predict both immediate and long-term response to cardiac resynchronization therapy (CRT). This expands the capabilities of echocardiography to noninvasively quantify mechanical LV dyssynchrony, which is becoming increasingly important in selecting patients for CRT [27].

Moreover, in evaluating longitudinal dyssynchrony, radial strain estimation may be complementary and even preferable. The temporal relationship of speckle-tracking radial strain demonstrated a strong correlation with corresponding measurements obtained using tissue Doppler radial strain in both the anteroseptal and posterior regions, thus enabling the assessment of dyssynchrony. Speckle-tracking strain was capable of ascertaining the timing of numerous sites that tissue Doppler was incapable of determining due to its independence from Doppler angle [27].

Myocardial work analysis has become a useful method for evaluating ventricular function, including identifying and describing ventricular dyssynchrony. Multiple software packages are accessible for the analysis of myocardial work, providing a range of metrics to assess ventricular mechanics. Several widely used software tools for evaluating ventricular dyssynchrony include EchoPAC (GE Healthcare, Chicago, IL, USA), Syngo.Via (Siemens Healthineers, Erlangen, Germany), and CVI42 (Circle Cardiovascular Imaging Inc., Calgary, AB, Canada).

Myocardial work analysis parameters include the following:

- The Global Myocardial Work Index (GWI) quantifies the overall mechanical work carried out by the ventricles throughout the cardiac cycle. The calculation involves determining the total area beneath the curve representing myocardial work during the entire ejection period;
- Segmental Myocardial Work: This analysis evaluates regional differences in myocardial work within the ventricles. This analysis offers valuable information on dyssynchrony by comparing work indices among various cardiac segments;
- Systolic Myocardial Work: This pertains to the effort exerted by the ventricles during the contraction phase of the heartbeat. The measures included are systolic work index (SWI) and peak systolic myocardial work (PSMW);
- Diastolic Myocardial Work: This assesses the effort exerted during the relaxation and filling of the ventricles. Parameters such as diastolic work index (DWI) and peak diastolic myocardial work (PDMW) are used in this context;
- Time-to-Peak Myocardial Work: This metric quantifies the timing at which peak work is achieved in various segments of the myocardium. Dyssynchrony is defined as the presence of delays in the time it takes for different areas of the ventricles to reach their peak work;
- Work Efficiency: This assesses the ratio of myocardial work to myocardial oxygen consumption, offering insights into ventricular performance and efficiency.

These software packages employ sophisticated algorithms to examine the deformation and mechanics of the heart muscle using imaging data. This allows clinicians to evaluate ventricular dyssynchrony without the need for invasive procedures. The criteria offered by these instruments assist in quantifying the extent of dyssynchrony and provide guidance for therapeutic decision-making, specifically in the treatment of heart failure patients who are suitable candidates for CRT.

### 2.3. Cardiac Magnetic Resonance

**Figure 5:**
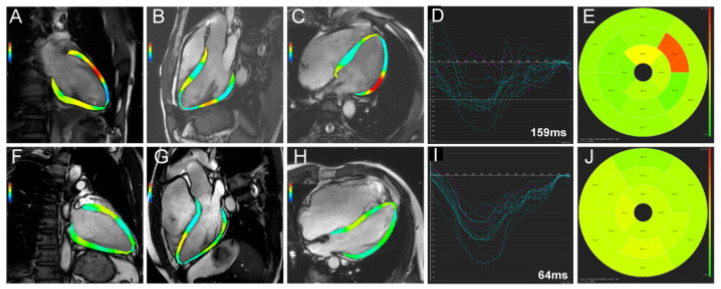
Time-to-peak longitudinal strain of the 16 segments. Images (**A**–**J**) represent different image acquisition incidents (Photo extracted from Song Y. et al., Left Ventricular Longitudinal Dyssynchrony by CMR Feature Tracking Is Related to Adverse Prognosis in Advanced Arrhythmogenic Cardiomyopathy. Front Cardiovasc Med. 2021 Oct 11;8:712832) [30].

Cardiac magnetic resonance (CMR) is a prospective alternative to echocardiography for guiding CRT by providing detailed information regarding the function of the left ventricle (LV), the presence of dyssynchrony, or scar tissue. In contrast to nuclear imaging, which captures multiple parameters in a single, reproducible scan, CMR offers superior spatial resolution without the use of ionizing radiation [28]. Feature tracking (FT), a nascent tissue tracking methodology assessed via conventional cardiac magnetic resonance (CMR) sequences, employs strain, strain rate, torsion, and dyssynchrony measurements to quantify myocardial motion and deformation, as seen in Figure 5. Having the capability to measure all cardiac chambers, including the challenging regions of thin-walled atria and the right ventricle, strain analysis via this method provides additional information beyond conventional global and segmental functional analysis, as acknowledged in the contemporary literature [29].

High spatial resolution, an outstanding signal-to-noise ratio, and dependable wall motion tracking are all characteristics of cardiac MRI. Dyssynchrony measurement, specifically tagging or strain imaging techniques such as displacement encoding with stimulated echoes (DENSE), has demonstrated potential in forecasting the response to CRT. However, its application is restricted to the scientific community as a result of the intricate imaging acquisition process and specialized postprocessing required [31].

The correlation between echocardiographic dyssynchrony indices, including septal flash and myocardial activity distribution, and a favorable response to CRT therapy has been substantiated by research [32,33,34]. On the contrary, a reduced response rate has been associated with the existence of myocardial scarring, particularly in the posterolateral wall or septum of the left ventricle [35,36]. Since the restoration of septal function is a key mechanism in the response to CRT, it is critical to identify septal viability. While cardiac magnetic resonance imaging (CMR) and echocardiography may work in tandem to detect dyssynchrony, a prospective multicenter study identified septal LGE as a significant ischemic predictor of non-response to CRT [37].

### 2.4. New Diagnostic Techniques

#### 2.4.1. Vectocardiography

A number of techniques for evaluating electrical dyssynchrony in order to select patients for cardiac rehabilitation therapy (CRT) have been devised in the last decade. An example of an approach is the three-dimensional (3D) vectorcardiogram (VCG), which quantifies the electrical impulses of the heart as a vector loop. The 3D area of the VCG QRS- (QRS-area) and T-wave loop (T-area) is believed to represent the unopposed electrical forces that occur during ventricular depolarization and repolarization, respectively. The QRS-area, derived from the reconstructed vectorcardiogram (VCG), is now the most often reported VCG measure for selecting individuals who will benefit from cardiac resynchronization therapy (CRT). Research has demonstrated that a QRS-area greater than or equal to 98 μVs is a more effective indicator of echocardiographic CRT result when compared to QRS length and LBBB shape. A QRS area greater than or equal to 109 μVs was found to be a more effective predictor of clinical and echocardiographic outcomes compared to QRS morphology and QRS length. Additionally, it was the only electrocardiographic factor that independently correlated with the clinical outcome. A Danish study conducted retrospectively found that a QRS area of 95 μVs or less was linked to a reduced clinical outcome. Additional VCG parameters that focus on repolarization instead of activation have been suggested as potential predictors of CRT outcomes. Nevertheless, the exact meaning of QRS-area in relation to the heart remains uncertain. Additional investigation into the correlation between QRS-area and intra-cardiac electrical features has the potential to yield useful insights into the underlying mechanisms. In summary, VCG QRS-area shows potential as a quantitative parameter that could outperform traditional criteria for selecting CRT [38].

#### 2.4.2. Ultra-High-Frequency ECG

The ultra-high-frequency ECG (UHF-ECG) approach is an advanced tool used to evaluate electrical dyssynchrony in cardiac rehabilitation treatment (CRT). Signals in sixteen frequency bands are analyzed from eight precordial leads, while ECG signals are recorded at a high sampling rate and with a band width of up to 1500 Hz. The technique quantifies ventricular electrical delay (e-DYS) and has been employed in clinical investigations to investigate ventricular activation patterns in various ways of CSP. UHF-ECG necessitates a specific device to capture high-frequency potentials. However, its ability to evaluate and visualize dyssynchrony in real time makes it especially advantageous during the implantation and optimization of CRT [38].

#### 2.4.3. Three-Dimensional Echography

The primary objective of echocardiography is to quantify intraventricular dyssynchrony, which is essential for determining the timing of regional LV mechanical contraction and decreasing the proportion of patients who do not respond to CRT therapy. Based on the American Heart Association (AHA) standard model, intraventricular dyssynchrony is assessed by segmenting the left ventricle into 16 or 17 segments. For each segment, the duration between the R wave on the electrocardiogram and the minimal systolic volume is computed; the systolic dyssynchrony index is the standard deviation of these regional times [39]. While findings regarding LV lead placement and remodeling are inconsistent, some studies imply that CRT therapy is beneficial for patients with a higher systolic dyssynchrony index [40,41,42,43]. By bypassing the technical limitations of the systolic dyssynchrony index acquired during cardiac systole and providing a comprehensive assessment of all LV mechanical deformation components, 3D speckle tracking surpasses the demands of tomographic imaging. By quantifying myocardial strain—a high LV lateral to septal work difference—this method can be utilized to forecast the CRT response [44].

In addition, the parameter 3D area strain, which includes both longitudinal and circumferential deformation, shows great potential in the quantification of LV dyssynchrony. Nevertheless, this technique is incompletely validated on account of its comparatively reduced temporal resolution, which is particularly problematic in dilated cardiomyopathy [40].

#### 2.4.4. Nuclear Imaging

Although radiation-free methods such as transthoracic echocardiography and CMR can be utilized to assess mechanical dyssynchrony, nuclear modalities present notable benefits in this regard. With no contraindications for the use of radiopharmaceuticals, SPECT distinguishes itself through its rapid acquisition, low variability, and ability to inform on both contractile function and perfusion [42,43].

Stress tests are frequently used to detect mechanical dyssynchrony, which is particularly susceptible to underestimation at rest, particularly in patients with coronary artery disease [45]. Subsequent to the stress test, however, post-stress measurements were performed, which account for the majority of the available data. Therefore, contractility disturbances, which are frequently detected through increased mechanical dyssynchrony indices, could potentially indicate myocardial stunning. However, it is important to note that this phenomenon is time-dependent and may resolve by the time perfusion imaging is performed [46,47].

All dyssynchrony parameters derived from SPECT in the study of ischemic patients by Legallois et al. exhibited a decreasing trend as dobutamine levels increased, indicating a greater degree of synchronous mechanical activation during rest [48]. Stress-gated blood pool SPECT (GBPS), which is independent of myocardial wall contouring, may assist in overcoming the potential for errors that may occur during the assessment of mechanical dyssynchrony when extended perfusion defects are present [49,50].

### 2.5. Novel Diagnostic Techniques for Improved Patient Selection for Cardiac Resynchronization Therapy (CRT)

Optimizing patient selection for cardiac resynchronization therapy (CRT) is essential for improving treatment outcomes and minimizing needless procedures. Novel diagnostic tools seek to enhance the precision of evaluating possible candidates for CRT by selecting patients who are most likely to get therapeutic benefits, as seen in Table 1.

#### 2.5.1. Non-Invasive Three-Dimensional Electrical Activation Mapping (ECGI)

Non-invasive three-dimensional (3D) electrical activation mapping, known as electrocardiographic imaging (ECGI), is a novel method that involves using a dense array of body surface electrodes placed around the patient’s torso. This method also utilizes the patient’s specific heart and torso geometry, which is obtained from computed tomography (CT) or magnetic resonance imaging (MRI). By combining these elements, it is possible to reconstruct the electrical activation of the epicardium or both the epicardium and endocardium in a single heartbeat without the need for invasive procedures [51].

Electrocardiographic imaging has shown the capability to evaluate the complete sequence of ventricular activation with a strong association to the invasive contact mapping technique. This is shown in native rhythms with large QRS, bundle branch block, and paced rhythms. Within the context of CRT, ECGI has demonstrated the ability to forecast both immediate and long-term reactions by evaluating the first electrophysiological activation of the left ventricle and the extent of ventricular dyssynchrony. The distance d_p_ between the latest electrically activated LV site and the LV pacing site predicts CRT response. The optimal cut-off point analysis showed that a value of dp = 47 mm divided the responder and non-responder groups to provide the best balance between sensitivity and specificity [51].

#### 2.5.2. Virtual Pacing of a Patient’s Digital Twin

A digital twin (DT) is a computer model that represents the patient’s heart by incorporating a collection of measurements specific to the patient. As a result, the DT can serve as a platform for assessing the effectiveness of therapeutic approaches and explains the particular pathophysiology of the patient. An algorithm that generates a DT using LV strain and volume measures was established based on the diagnostic utility of myocardial strain measurements in patients with dyssynchronous HF.

Reducing septal to lateral work imbalance in the DT via virtual pacing can predict LV reversal remodeling in patients following CRT; this may serve as an additional criterion for selecting patients with heart failure who require CRT [52].

## 3. Clinical Impact

The negative effects of LBBB on cardiac function are seen acutely, including not only myocardial dysfunction but also mitral regurgitation as well as effects on the right ventricle [51]. The presence of systolic dysfunction LBBB arises from the lack of coordination in the contractions of the left ventricle. Specifically, the septum, which is activated earlier than the rest of the ventricle, contracts out of sync and pushes blood towards the stretched lateral wall. After contracting forcefully, the lateral wall pushes blood towards the stretched septum, effectively absorbing energy. The loss of a large percentage of the septal contribution in LBBB imposes a considerable burden on the left ventricle’s lateral wall, leading to unfavorable remodeling in patients with heart failure [53,54].

In cases of LBBB and non-ischemic cardiomyopathy, the septum’s clockwise pressure–strain loop displays a lack of effectiveness in contraction. This is shown by systolic lengthening and negative (inefficient) work. In contrast, the lateral wall exhibits a normal counterclockwise rotation and systolic shortening [55]. Numerous individuals diagnosed with LBBB have a decrease in blood flow to the septum, even when there is no presence of coronary artery disease. This is caused by the relative lengthening of the contraction phase of the septum during exercise-induced rapid heart rate [56].

During regular LV electrical activation, the papillary muscles contract before the LV walls, which results in the tightening of the chordae tendinae. This tightening occurs before the production of LV pressure and serves to restrict the flow of blood back into the left atrium. LBBB interrupts the synchronized activity of the heart and is one of the factors that leads to mitral regurgitation [57]. In individuals with dilated cardiomyopathy, there is an elevation in the stresses exerted on the mitral leaflets, resulting in a decrease in the competence of the mitral valve. Consequently, the appropriate closure of the mitral valve becomes more reliant on the early systolic left ventricle. The reduced rate of left ventricular pressure in LBBB leads to a decrease in the force that closes the mitral valve, which is a significant factor in the development of mitral regurgitation in cases of cardiac dyssynchrony [58].

Regarding diastolic function, ventricular dyssynchrony leads to an elongation of the left ventricle’s pressure decay and prolongs the time it takes for the ventricle to relax without any change in volume, hence reducing the time available for left ventricular diastolic filling. Tachycardic pacing results in diastolic stiffening as a consequence of incomplete left ventricular relaxation caused by an abbreviated filling time [59,60]. Similar to other patients with heart failure and lower ejection fraction, individuals with ventricular dyssynchrony will undergo a compensatory increase in left ventricular (LV) filling pressures [24]. At present, there is no evidence establishing a connection between decreased left ventricular diastolic function and heart failure symptoms in patients with left bundle branch block (LBBB). It is likely that these patients need higher left atrial pressure to achieve equal quantities of ventricular filling [61].

Moreover, the restoration of synchronous activation has a substantial effect on the energy usage of heart muscle cells. This is because it reduces excessive stretching, which in turn helps to restore normal cellular metabolism and function [61].

## 4. Treatment Strategies

### 4.1. Cardiac Resynchronization Therapy (CRT)

The simultaneous presence of left bundle branch block (LBBB) and intraventricular conduction disorders is linked to a 30% increased risk of death from any cause. Consequently, ventricular dyssynchrony represents a critical therapeutic target for HFrEF patients [61].

**Figure 6:**
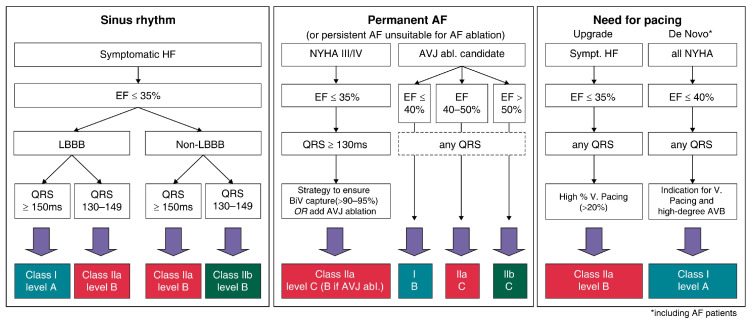
Cardiac resynchronization indications from ESC 2021 pacing guidelines (Photo extracted from Kenneth A. Ellenbogen et al. The evolving state of cardiac resynchronization therapy and conduction system pacing: 25 years of research at EP Europace journal, *EP Europace*, Volume 25, Issue 8, August 2023, by permission of Oxford University Press) [63].

As per present recommendations, individuals who have HFrEF and an abnormal QRS (complex LBBB morphology and/or QRS duration > 130 ms) are suitable candidates for CRT, as seen in Figure 6. Similarly, due to the similarity between the activation sequence in RV pacing and that of LBBB, CRT is recommended for patients who are RV-paced for a substantial portion and have LVEF < 35%, as well as patients with LVEF < 40% and high-degree AV block who require ventricular pacing [61].

The notion of “non-responders” is largely exclusive to the domain of CRT. Nonetheless, it is common knowledge that treatment responses are seldom perfect. Three categories have been proposed in a recent joint position statement by the European Association of Cardiovascular Imaging, the Heart Failure Association, and EHRA: disease progression; partial remission; and complete remission or cure in response to CRT [62].

#### 4.1.1. Challenges in Conventional CRT

Challenges in traditional cardiac resynchronization therapy (CRT) can occur due to limitations in delivering left ventricular (LV) pacing caused by the structure of the cardiac veins (such as loss of electrical signal, displacement of the pacing lead, suboptimal positioning of the lead, or pacing of the diaphragm). These challenges can be overcome by implanting a quadripolar LV lead or an epicardial LV lead or by using endocardial LV pacing. Additionally, this approach may provide a resolution for suboptimal RV/LV depolarization that may occur due to factors such as delayed propagation of myocardial impulses in diseased tissue, prolonged latency from stimulus to QRS, or RV anodal capture [64].

In the event of inter/intra-atrial block, delayed atrial sensing, or absence of LV preexcitation due to fusion, AV junctional ablation may be considered as an alternative method that precludes the need for fused/adaptive CRT. Ablation may be implemented as a feasible solution in the event that contending premature ventricular contraction or atrial fibrillation derail CRT [64].

#### 4.1.2. Predictors of Therapeutic Response

Villegas-Martinez et al. undertook a study to investigate pulse arrival time (PAT) as a non-invasive indicator of the immediate and long-term responsiveness to CRT. Patients with a long-term response to CRT exhibited acute improvement as measured by a reduction in PAT duration; however, patients who did not respond did not experience a reduction in PAT duration. PAT could be utilized as an appealing and non-intrusive technique for evaluating the immediate reaction to CRT during the process of implantation and optimization of CRT [65].

The time between the electrical onset and the foot of the peripheral pressure waveform was utilized to calculate PAT. This value is equal to the sum of two factors: the pre-ejection period and the speed-dependent travel time of the pressure wave from the aortic valve to the peripheral artery. Therefore, unless there is a significant alteration in blood pressure, the pulse wave velocity is expected to stay consistent during CRT testing. The aforementioned investigation revealed that the precision of PAT in estimating relative changes in LV function was similar to that of Td, a substitute for PEP, which has been demonstrated to enhance the optimization of CRT [65].

Early detection of disease modification prior to implantation of CRT might assist in selecting appropriate patients, enhancing responder rates, and encouraging the adoption of CRT. The study conducted by Odland et al. aimed to test the hypothesis that the shortening of time-to-peak left ventricular pressure rise (Td) with cardiac resynchronization therapy (CRT) can be used to predict long-term volumetric response. The researchers observed that Td has the potential to serve as a marker for predicting long-term volumetric response in candidates for CRT [66].

### 4.2. His Bundle Pacing

His bundle pacing is characterized by the direct activation of every fiber of the atrioventricular bundle during capture. The distal atrioventricular node delineates this segment of the heart’s conduction axis proximally, while the division of the His bundle into the right bundle branch (RBB) and left bundle branch (LBB) demarcates it distally. His bundle potential to QRS interval at the pacing site being >=35 ms, the pacing lead being positioned near the tricuspid valve summit, either on the atrial or ventricular side of the tricuspid annulus, and the presence of HBP capture criteria are all distinctive features of HBP. Distal His bundle pacing from deep within the septum may occur in certain patients [63].

**Figure 7:**
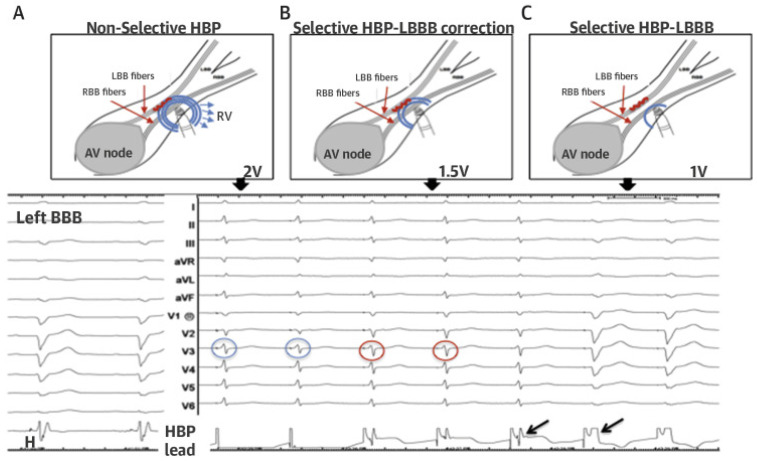
Longitudinal Dissociation Within the His Bundle (Photo extracted from Vijayaraman P. et al., ACC’s Electrophysiology Council. His Bundle Pacing. J Am Coll Cardiol. 2018 Aug 21;72(8):927–947) [67].

Maintaining electromechanical synchrony (interventricular and intraventricular) is the primary clinical benefit of HBP. The electromechanical synchrony remains unaffected in S-HBP with normal HPS conduction, as the duration and morphology of the paced and native QRS remain unchanged. With enhanced mechanical synchrony, electrical synchrony would presumably return to normal in S-HBP with underlying BBB and complete correction. The potential for ventricular dyssynchrony in NS-HBP, which combines pre-excitation of the septal myocardium and conduction through the HPS, is a matter of debate. When the His–Purkinje conduction reaches the myocardium, NS-HBP generates a pseudo-delta wave that abruptly transforms into a precipitous dV/dT, with a timing approximating the HV interval, resulting in a QRS widening of duration ≤HV interval as seen in Figure 7 [67].

Comparing NS-HBP to S-HBP or intrinsic activation, the LV total activation time does not vary substantially. Early activation, on the other hand, occurs in the LV epicardium during biventricular pacing, which is an entirely distinct pattern of activation [67].

Hemodynamics during HBP are favorable in comparison to right ventricular pacing (RVP), according to acute electrophysiology investigations. Comparing His and RA pacing in 31 patients with narrow QRS undergoing an electrophysiological study, Ji et al. found no significant difference in LV circumferential strain, radial strain, twist, and mechanical dyssynchrony; however, RV outflow tract and RV apical pacing exacerbated these parameters. Whether HBP can take the place of LV pacing in individuals who qualify for CRT may be determined by comparing it to LV or biventricular pacing [67].

An acute temporary HBP versus biventricular pacing study conducted by Sohaib et al. involved fourteen patients who presented with systolic HF, prolonged PR > 200 ms, and narrow QRS < 140 ms or RBBB [68]. These patients exhibited enhanced acute hemodynamic function as evidenced by a 4 mm increase in blood pressure (for both biventricular pacing and HBP with AV shortening/optimization) compared to those who received intrinsic rhythm. Biventricular and LV-only pacing improved systolic function and LV synchrony at individually optimized AV delays, whereas His-LV pacing enhanced indexes at all AV delays in comparison to AAI pacing [67].

### 4.3. Left Bundle Branch Pacing

The term “LBBP” refers to the ability to capture both the LBB and the LV septal myocardium at a low output (<1.0 V with a 0.5 ms pulse width). The ventricular pacing lead is positioned 1–2 cm apical and ventricular to the distal His bundle region within the interventricular septum in LBBP, in the vicinity of the LB or its branches [62].

The criteria for LBB capture are not uniformly defined and established in the early stages of LBBP. As the application and investigation of LBBP continue to expand, the precise definition of LBB capture is progressively solidifying. In general, LBBP can be classified into two types: selective LBBP (S-LBBP), which captures the LBB exclusively, and non-selective LBBP (NS-LBBP), which captures both the LBB and the adjacent local myocardium [61].

**Figure 8:**
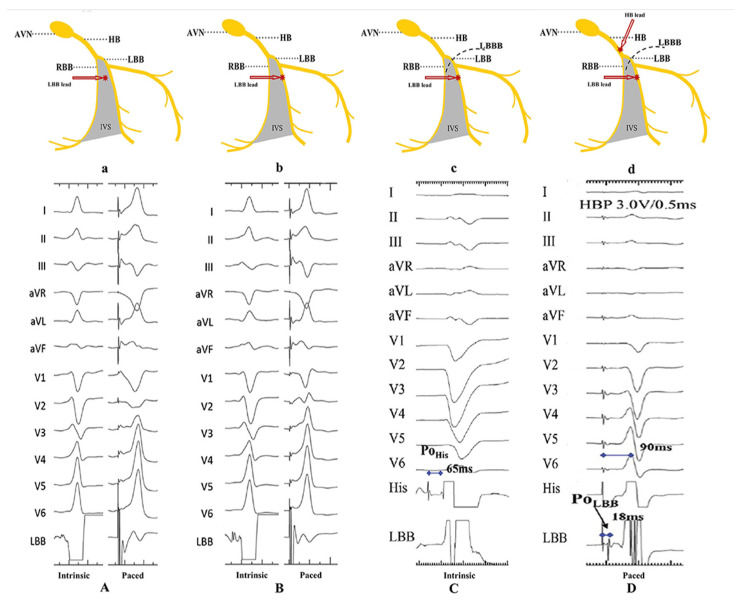
Recording of the LBB potential. (**A**) The LBB potential can be recorded when the pacing lead helix is approaching the LBB. (**B**) The LBB potential becomes larger when the lead is closer to or at the LBB. (**C**,**D**) The LBB potential cannot be recorded in patients with LBBB, unless LBB is corrected by HBP (9). AVN, atrioventricular node; HB, His bundle; LBB, left bundle branch; LBBB, left bundle branch block; RBB, right bundle branch; IVS, interventricular septum; PoHis, His potential; PoLBB, left bundle branch potential. (Photo extracted from Liu P., Wang Q., Sun H., Qin X., Zheng Q. Left Bundle Branch Pacing: Current Knowledge and Future Prospects. Front Cardiovasc Med. 2021 Mar 23;8:630399) [69].

LBBP is characterized by the following elements: (1) an RBBB pattern, (2) an LBB potential, (3) S-LBBP exhibiting distinct ECG changes and a discrete component on electrocardiogram, and (4) a stimulus to the LV activation time that is both constant and brief, irrespective of whether the pacing outputs are high or low. The characteristics of the indirect criteria for LBB capture are utilized to distinguish LBBP from LV septal pacing (LVSP), as seen in Figure 8. Therefore, LVSP is erroneously identified as LBBP in certain instances. Retrograde His bundle potential or anterograde left conduction system potentials were suggested by Wu et al. as a means to directly validate LBB capture and differentiate between LBBP and LVSP with greater precision [61].

This technique, nevertheless, is complicated and unsuitable for routine clinical application. Within this framework, the notion of LBB area pacing (LBBAP) has been suggested as an alternative to LBBP or LVSP, despite the absence of definitive proof of LBB capture [61].

### 4.4. Deep Septal LBBB Pacing

The technique known as deep septal LBBB pacing, which was devised by Huang et al. in 2017, involves positioning the lead trans-septally from the RV septum to the subendocardial LV septum in order to directly stimulate the LBB region. Aiming for a distance of 1–1.5 cm from the His recording site on the RV septum is the most advantageous location for fixation. The QRS morphology prior to fixation typically exhibits a w-shaped pattern with a notch at the lowest point of the QRS complex in lead V1. The paced QRS morphology transitions from an LBBB to an RBBB configuration as the lead is advanced into the septum. Significant QRS reduction, shortened LV activation time, improved LV ejection fraction, diminished LV dimensions, and decreased NYHA functional class were observed in two recent observational studies of LBPBP in HF patients with LBBB; LBBB and large LV end systolic diameter were not independent predictors of response. The success percentage of implantation in all the observational studies varied between 85% and 97%. This approach may be a good substitute for CRT because the left bundle stimulation threshold was consistently excellent, with a large R wave amplitude. Nevertheless, it is necessary to conduct randomized clinical trials in order to definitively establish this innovative pacing therapy as a primary choice [64].

### 4.5. HOT-CRT

Conduction abnormalities, such as distal LBBB or IVCD, present difficulties during implantation and follow-up of cardiac rehabilitation therapy (CRT). The anatomical structure of the heart and the branches of the coronary sinuses are the limits of numerous attempts to identify the optimal pacing site. Electrical resynchronization is a valuable tool for predicting the efficacy of cardiac resynchronization therapy (CRT). An elevated proportion of RV pacing has a negative impact on the effectiveness of therapy. Atrial fibrillation is prevalent in patients with heart failure, and traditional cardiac resynchronization therapy (CRT) systems are ineffective in addressing this problem. In order to enhance CRT, an extra lead can be utilized in the His bundle, which facilitates uniform activation of the right ventricular septum during atrial fibrillation. This technique is capable of rectifying intricate conduction defects, such as the presence of coexisting intraventricular conduction delays in individuals with progressive cardiomyopathy. HOT-CRT (His-optimized CRT) is an innovative pacing technique that enhances cardiac resynchronization therapy (CRT) by combining pacing from both the His bundle and the left ventricle (LV). This results in improved fusion pacing, which is less affected by the inadequate positioning of the LV due to anatomical factors [70].

Rickard et al. conducted a study that examined the impact of cardiac resynchronization therapy (CRT) on 865 individuals who had chronic right ventricular (RV) pacing. The study also compared the response to therapy in this group to a separate group of patients with no previous treatment (de novo cohort). The findings indicated that RV caused dyssynchrony, and even individuals with a dyssynchrony duration exceeding 200 ms benefited from CRT. Individuals with an EF < 35%, NYHA III, and QRS > 150 ms were included in the Multisite Stimulation in Cardiomyopathy (MUSTIC) research. Although the QRS width decreased, there were noticeable enhancements in the echocardiographic characteristics [71].

A study conducted by Vijayaraman et al. has shown that an extra His bundle lead is a potential choice for patients with atrial fibrillation who rely on cardiac resynchronization therapy (CRT). Patients with preexisting medical conditions would have found treatment with either purely His or only BiV to be ineffective or less than ideal. The recently released multicenter MELOS study presents left bundle branch region pacing as a viable alternative to His bundle pacing. The LOT-CRT technique demonstrates the efficacy of the HOT-CRT idea, showing that combined His and LV pacing may effectively address distal branch blockages and IVCD, which are difficult to rectify with RV/LV pacing alone [70,72].

## 5. Conclusions

In addressing the complex challenges of treating heart failure with reduced ejection fraction accompanied by conduction disorders such as left bundle branch block, the landscape of therapeutic interventions has significantly evolved. Cardiac resynchronization therapy stands as a cornerstone, particularly for those exhibiting specific electrocardiographic characteristics indicative of ventricular dyssynchrony. The advent of His bundle pacing and left bundle branch pacing further refines the approach, offering more physiological pacing modalities that promise enhanced outcomes by maintaining or restoring the natural sequence of ventricular activation.

Despite these advances, the heterogeneity in patient responses underscores the necessity for personalized treatment strategies, as seen in Table 2. The concept of HOT-CRT emerges as a pivotal innovation, amalgamating the benefits of CRT with the precision of His bundle or left bundle branch area pacing, aiming to optimize cardiac function in a subset of patients where traditional CRT might fall short.

Emerging diagnostic techniques for cardiac dyssynchrony, including vectorcardiography, ultra-high-frequency ECG, 3D echocardiography, electrocardiographic imaging, and virtual pacing of a patient’s digital twin, offer advanced precision in identifying optimal candidates for cardiac resynchronization therapy. By improving the accuracy of patient selection, these techniques aim to maximize the therapeutic benefits of CRT, thus potentially improving patient outcomes and optimizing the use of healthcare resources in treating heart failure.

The dynamic field of cardiac pacing continues to evolve, driven by technological advancements and deeper insights into cardiac electrophysiology. Future research, particularly randomized clinical trials, will be crucial in delineating the optimal therapeutic pathways for individuals with HFrEF and conduction abnormalities. Such endeavors will not only refine existing treatment algorithms but also potentially expand the candidacy for these life-enhancing therapies, ensuring that patients receive the most effective, tailored interventions. In summary, the journey from recognizing the detrimental impact of ventricular dyssynchrony to innovating and implementing sophisticated pacing strategies exemplifies the relentless pursuit of excellence in cardiac care, promising improved outcomes for patients grappling with complex cardiac conditions.

## Figures and Tables

**Table 1:** Main diagnostic techniques used in dyssynchrony.

Diagnostic Technique		Method	Cut-Off	Advantages	Disadvantages
1. ECG		QRS duration	≥150 msec		Insufficient for distinguishing between right- or left-sided conduction abnormalities and inter- versus intraventricular dyssynchrony
2. ECO	Opposing wall delay	TDI color peak velocity—4 chambers, PLAX	≥65 msec	Rapid, can be analyzed offline	Requires TDI color equipment; susceptible by passive tethering motion
	Maximum wall delay	same	≥100 msec	Complete detection of longitudinal dyssynchrony	Requires TDI color equipment; susceptible by passive tethering motion
	Yu index	TDI color (apical 2/4 chamber, PLAX)	≥33 msec	Same	Requires TDI color equipment; susceptible by passive tethering motion; time-consuming
	Delay in initiation of systolic velocity	Same	≥100 msec	Widely available	Not possible offline; affected by passive tethering motion; difficulty in acquiring
	Delay in longitudinal contraction	TDI strain (apical view)	-	Less affected by passive tethering motion	Specialized equipment, demanding technique
	Septal–posterior wall delay	M mode parasternal LV view	≥130 msec	Rapid, widely available	More affected by passive motion or tethering; akinesis provides difficulty
		Radial strain—parasternal mid-LV view—speckle tracking	≥130 msec	Speckle tracking may be applied; less affected by passive motion/tethering	Specialized equipment; assessing only radial dynamics
	Mechanical delay	Routine pulsed Doppler (RVOT/LVOT views)	≥40 msec	Widely available; advanced technique; highly reproducible	Affected by LV and RV function
	Longitudinal shortening as percentage GLS	Speckle tracking, apical view of LV	−9%	Waveforms can illustrate contraction delay and temporal dispersion in multiple myocardial segments; higher reproducibility	Errors in triggering, definition of region of interest; depends on preload, afterload, and heart rate; interobserver and intra-observer variability
	Myocardial wall shortening from inward movement of overall endocardial circumference GCS	Speckle tracking; mid-papillary LV short-axis view	−9%	Unchanged in patients with severe aortic stenosis; does not discriminate the contractility of different LV wall layers	Learning skills slower; lower reproducibility
	Transmural strain GRS	Speckle tracking; mid-papillary LV short-axis view		Unchanged in patients with severe aortic stenosis; does not discriminate the contractility of different LV wall layers	

**Table 2:** Treatment options for heart failure with LBB and EF < 35%.

Technique	Advantages	Disadvantages
Cardiac Resynchronization Therapy	Improve quality of life, reducing symptomatology Improves mechanical dyssynchrony Decreases cardiac workload and hospitalization	30–40% non-responders, including those with LV scar, suboptimal LV stimulation site, limited electrical and mechanical dyssynchrony Inability to stimulate severely diseased myocardium or myocardial scar without massive stimulus-to-QRS latency. Requires LV pre-excitation, often in conflict with delivery of ventricular pacing at optimal AV interval
His Pacing	Most physiological variant Prevents RV pacing induced cardiomyopathy In patients with Afib: can be a better method for delivering pacing since the lead can be positioned distal to the ablation site Alternative technique for CRT therapy can significantly shorten QRS duration and restore normal intrinsic activation patterns in patients with ventricular conduction delays.	Narrow target zone—lengthy procedure, fluoroscopic time longer High capture thresholds, late follow-up can lead to premature battery depletion and replacement of generator. Low sensed R wave amplitude, which can cause oversensing of atria or His signals and undersensing ventricular signals. In patients with infra-hisian block, success rate is lower May not correct BBB in all patients and may be combined with uni- or biventricular fusion pacing.
LBBB Pacing	Low and stable thresholds Wider area of LBB fibers on the sub-endocardium Lead stability Adequate safety margin for ablation in patients undergoing AV junction ablation	Lack of evidence regarding definition of left conduction system capture and left ventricular septal capture
Deep Septal LBBB Pacing	Relative physiological ventricular activation Reduces electric dyssynchrony Preserves LV pump function	Procedural challenges
HOT-CRT	Improved echocardiographic and clinical improvement in advanced HF patients Patients with Afib, the atrial port can provide an excellent opportunity to use His synchronous LV pacing. Improves electrical dyssynchrony beyond BiV or multipoint pacing	Patients in sinus rhythm—challenge to incorporate the fourth lead without compromising other device functions.

## Data Availability

Not applicable.
